# Precision Medicine and the Institutional Review Board: Ethics and the Genome

**DOI:** 10.31486/toj.19.0098

**Published:** 2020

**Authors:** Marc R. Matrana, Bob Campbell

**Affiliations:** ^1^ Ochsner Cancer Institute, Ochsner Clinic Foundation, New Orleans, LA; ^2^ The University of Queensland Faculty of Medicine, Ochsner Clinical School, New Orleans, LA; ^3^ Ochsner Institutional Review Board, Ochsner Clinic Foundation, New Orleans, LA

**Keywords:** *Ethics*, *ethics committees*, *genetic privacy*, *genomics*, *precision medicine*, *research*

## Abstract

**Background:** Clinical research studies often integrate precision medicine technologies and techniques, offering novel treatment opportunities for patients but also posing significant challenges for regulatory authorities and local institutional review boards (IRBs) as they attempt to protect patient safety and privacy.

**Methods:** We review the basics of precision medicine and discuss how IRBs are addressing new challenges associated with the era of precision medicine.

**Results:** Precision medicine trials rely on genomic testing for inclusion criteria and investigational drug therapy choices. The vast amounts of complex information that can be obtained from basic genetic sequencing tests must be stored, analyzed, and interpreted, creating challenges for clinicians, researchers, and regulatory staff who are concerned with complex ethical, security, and legal issues surrounding patients’ personal genetic data in the digital age. All members of the IRB face a rapidly changing environment. The traditional areas of primary concern, such as patient privacy, terminology, and financial benefits, have been joined by issues associated with precision medicine, such as accelerated US Food and Drug Administration drug approval, multiple informed consent form modifications, increasing length and complexity of informed consent forms, and participant genetic privacy. The challenge to the IRB is to remain focused on the prior areas of significance while also adapting the evaluation process to the novel science of precision medicine.

**Conclusion:** In this era of exponentially increasing big data and easy-to-access genetic sequencing data, IRBs will be tasked with adapting their processes and adjusting to the new technology and its corresponding complexities. Such adaptation has always been required of IRBs, but now it will need to occur rapidly as technology and data analysis capabilities accelerate.

## INTRODUCTION

Precision medicine, genomics, and molecular medicine can have major impacts on health outcomes for patients and are changing how providers approach and select treatments for patients. Today, clinical research studies often integrate precision medicine technologies and techniques that offer advanced treatment opportunities to patients but also present significant challenges for regulatory authorities and local institutional review boards (IRBs) as they attempt to protect patient safety and privacy in an era of big data and genetic sequencing.

## DEFINITION AND BASIC CONCEPTS OF PRECISION MEDICINE

In broad terms, precision medicine is the customization of treatments at the individual patient level. In practice, precision medicine is a catchall term that encompasses a wide range of genomic and molecular technologies that pair individual patients with the most effective, least toxic therapies. To understand these technologies, it is important to start from the beginning.

Oswald Avery discovered DNA in 1944, and Watson and Crick reported its double-stranded structure in 1953.^1,2^ Since then, our knowledge of the structure and function of the human genome has increased exponentially. The human genome project completed the first full sequencing of the human genetic code in 2003, an endeavor that took more than a decade and cost $2.7 billion. Today, we can routinely complete similar sequencing in less than a week at a cost of approximately $1,000 to $3,000.^3^

Precision medicine relies on emerging genetic technologies that allow us to read the genetic code of individuals. At the most basic level, each cell contains a nucleus, which contains 23 pairs of chromosomes. These chromosomes are made up of long, double-stranded DNA that can be further divided into genes, the common unit of inheritance. Portions of this DNA are transcribed into RNA, which is translated into amino acids, the building blocks of proteins. Strings of amino acids fold in specific ways to form proteins that form cells and tissues and create enzymes that catalyze all essential functions of life. A point mutation—a change in a single molecule in the DNA coding sequencing—can have devastating effects on an individual's health. Once these mutations are identified, treatments that precisely target the root causes of illness can be selected, providing better care than the standard of care and, in some cases, potentially preventing disease.

As knowledge in this area of medicine expands and access to molecular testing becomes easier and cheaper, precision medicine is becoming mainstream. The largest and most progressive healthcare systems are creating departments or systemwide institutes focused on integrating genomic and other molecular testing into the daily workflow of providers, as well as providing interpretative support and education.

Emerging genomic technologies offer promise in every field of medicine, but the impact has been greatest in oncology. The survival time of patients with cancer can be significantly increased when the underlying genetic drivers of the cancer are uncovered.^4^ The Ochsner Precision Cancer Therapies Program coordinates genetic sequencing for all cancer patients, coordinates the statewide Louisiana Molecular Tumor Board, and oversees all early-phase clinical trials, including trials that match patients who have rare genetic mutations that drive their tumors to experimental drugs designed specifically to target those mutations.

## GERMLINE TESTING

Germline testing is testing for hereditary genes—genes that run in families. By identifying families that harbor certain genetic mutations that predispose them to disease, we can more accurately screen for, and in some cases, prevent certain illnesses. For example, for patients found to have hereditary nonpolyposis colorectal cancer, intense screening for colorectal cancer, ovarian cancer, and bladder cancer is recommended.

## NEXT GENERATION SEQUENCING AND BEYOND

Testing individual genes can be cumbersome and costly, especially when multiple genes need to be tested in a single patient sample. Next generation sequencing (NGS) allows for a curated panel of dozens or even hundreds of genes at one time from a small sample of tissue, blood, or urine. NGS is becoming the standard of care for patients with advanced cancer, especially for patients who have failed standard of care options. The Ochsner Precision Cancer Therapies Program offers comprehensive 500+ gene panel testing free of charge to all patients with advanced solid tumors and lymphomas through a partnership with Strata Oncology, a company that offers testing and clinical trials at a select network of cancer centers.^5^ The academically inclined partnership and network are unique because of the array of industry-sponsored precision medicine trials made available to the cancer treatment centers in the network and the use of pharmaceutical industry funding to cover the costs of patient screening and genetic sequencing, rather than passing those costs along to insurers or patients as is usually done.

NGS can be life changing for some patients. The testing sometimes reveals specific actionable genetic mutations that allow clinicians to pair patients with specific therapies that target underlying genetic mutations—therapies that may be more effective and less toxic than standard of care options. For example, patients with cancer who are refractory to standard therapies and who are found to have a rare neurotrophic tyrosine receptor kinase (NTRK) mutation can be treated with an NTRK inhibitor, such as larotrectinib, regardless of their cancer type. This drug, and drugs like it, achieve an almost miraculous efficacy, with clinical response seen in almost every patient and with far fewer side effects than most traditional cancer treatments.^6^

However, NGS is associated with challenges, principally tumor heterogeneity and tumor evolution. Every cell in a tumor arises from one aberrant progenitor cell. As daughter cells divide again and again, some of the cells will evolve differences and pass these differences on to *their* daughter cells. This process creates a kind of chimerism in which a single tumor may harbor cells that look different on a genetic level. Numerous studies document the presence of two adjacent cancer cells that came from the same progenitor cell but are different from one another. Likewise, tumors change over time. When performing NGS on an archived sample of a tumor, we cannot guarantee that the tumor in vivo has not changed since the biopsy was taken. This possibility necessitates the question of whether fresh biopsies need to be obtained for testing and, if so, how often and when.^7-9^

NGS is also associated with challenges in clinical decision making. Often, sequencing reveals mutations for which no commercially available therapy or experimental drug is available, or sequencing discovers mutations in a tumor type for which a corresponding target drug has not been studied. For example, a mutation for which a targeted drug is available may be found in breast cancer, but the drug has only been approved for use in lung cancer.

To try to make sense of this complex data and to answer these challenging questions are the reasons the statewide Molecular Tumor Board was created. Experts from various fields meet to discuss the most challenging patient cases and complicated sequencing results.

Beyond clinical conundrums, NGS is associated with financial challenges. The cost of NGS is dropping dramatically, but some insurers still do not cover it. Similarly, many insurers do not pay for targeted drugs unless the drug has been tested and approved for the specific type of tumor the patient has. For instance, an insurer might refuse to pay for an ALK inhibitor for a patient with colon cancer, even though the patient has the same mutation that drives lung cancer, but the drug is only approved for lung cancer tumors. Dedicated reimbursement specialists can help with these situations, and more health systems are hiring dedicated reimbursement personnel or even teams devoted to precision medicine.

Beyond NGS, whole exome sequencing takes genetic testing a step further by testing the coding sequence of all genes in a patient's entire genome. One step further is whole genome sequencing, which reads a patient's entire genome.

## BEYOND GENOMICS

Although genetic testing is currently at the heart of precision medicine, emerging techniques in proteomics, the large-scale study of proteins throughout the body, metabolomics, the large-scale study of metabolites, and microbiomics, the study of microorganisms such as bacteria found in the body, are expected to have profound effects on the practice of medicine in the future. By analyzing and altering molecules and microorganisms within the patient's body, clinicians may be able to detect diseases much earlier than they can at present and provide better prevention and treatment.^10^

## PHARMACOGENOMICS

Pharmacogenomics is an emerging science focused on the intersection of pharmacy and genomics. This discipline studies the effects of a person's genes on his or her response to medications. Some individuals who harbor certain genetic mutations have serious side effects to some medications. For example, patients with mutations leading to dihydropyrimidine dehydrogenase deficiency are known to develop severe and even life-threatening adverse events from treatment with 5-fluorouracil.^11^ By identifying these genetic differences before treatment is begun, we can avoid giving certain patients drugs that will likely harm them but may be harmless for patients who do not have those genetic mutations. Likewise, we know that certain patients respond better to one drug vs another, and pharmacogenomics can permit better matching between medications and patients in the attempt to achieve maximal benefit. More drugs with effects linked to testable genetic variations are being identified. For example, the information provided by a quick buccal swab can now identify which antidepressant will be most effective for a patient, eliminating the necessity of initiating a traditional trial of different antidepressants for periods of weeks to months.

## BIOINFORMATICS AND BIG DATA

Vast amounts of complex information can generally be obtained from basic genetic sequencing tests. The human genome contains more than 20,000 genes made up of 3 billion base pairs. Each gene can have a near limitless number of allelic variations that may lead to a large number of different individual phenotypic expressions in a patient.^12^ Sequencing even a small panel of genes provides an immense amount of data that must be stored, analyzed, and interpreted, creating challenges for clinicians, researchers, and regulatory staff who are concerned with complex ethical, security, and legal issues surrounding patients’ personal genetic data in the digital age. Information discovered through genetic sequencing can have consequences for patients. For example, a patient who is genetically at a higher risk for a certain disease may face certain types of discrimination or have psychological effects from knowing that he or she may develop a serious illness.

## PRECISION MEDICINE AND RESEARCH

Benefits of precision medicine are being realized in the clinical setting because of research. As new technologies have been developed, new trial designs—umbrella trials, basket trials, and adaptive trials—have been developed to align with the evolving science.

### Umbrella Trials

Umbrella trials are studies of many different mutations across different patients who have a single type of cancer. These studies typically have numerous treatment arms. Patient subjects are assigned to a treatment arm based on the specific molecular makeup of their cancer. The Lung-MAP trial (NCT02154490) is an example of an umbrella trial in which patients with a specific type of lung cancer are assigned to a treatment arm based on their cancer's underlying genetic mutations.^13^

### Basket Trials

Basket trials focus on one mutation and its corresponding treatment across a variety of tumor types. Compared to other trial designs, these studies have the potential to increase the number of patients who are eligible to receive certain drugs. An example of a basket trial is Loxo Oncology's single-arm NAVIGATE study (NCT02576431) in which patients with NTRK gene fusions across many solid tumor types received larotrectinib.^14^ This drug gained US Food and Drug Administration (FDA) approval based on the NAVIGATE study and two similar trials: the LOXO-TRK-14001 (NCT02122913) and SCOUT (NCT02637687) studies.

### Adaptive Trials

Adaptive trials allow for the evolution of a study as new data are discovered. By specifying certain statistical and design parameters at the outset, a single trial can morph over time. This design has many advantages. First, because data emerge rapidly in the field of precision medicine, traditional trials are often behind the therapeutic curve before they even start. In comparison, adaptive trials can be changed as the standard of care changes. Second, an adaptive trial can function as one overarching trial that can essentially last indefinitely as new treatments and arms are added or removed. The advantage is that laborious startup procedures only need to be done one time, and once the trial is open, it can address different treatment options and answer different questions as it changes over time. The National Cancer Institute MATCH trial (NCT02465060), a large multiarm study that combines aspects of basket and umbrella trials, is an example of an adaptive trial that evolved over time.^15^ The MATCH trial includes multiple evolving substudies, each covering a specific genetic mutation aimed across different tumor types.

## INSTITUTIONAL REVIEW BOARDS IN THE ERA OF PRECISION MEDICINE

The role of the IRB is to protect the rights and welfare of research subjects.^16^ The mission of the IRB, through its members, is to help researchers conduct important studies in a way that protects the rights and welfare of research participants.^17^ The IRB panels at Ochsner are comprised of scientists, nonscientists, and institutional and community members with different views, concerns, biases, and experiences that provide a multifaceted filter through which the IRB evaluates, debates, and decides on the disposition of research studies. This diverse membership allows the IRB to systematically evaluate each research study with perspectives from inside and outside the institutional healthcare arena. Each member has the opportunity to contribute his or her concerns and life experiences to the discussion.

The input from community members tends to focus more on the nonscientific aspects of the study and the individual rights of research subjects rather than on the scientific and statistical aspects of the trial. Issues such as the vocabulary used in consent forms, risks, benefits, protections of privacy, financial implications, descriptions of procedures, durations of participation in the study, and other areas of primary interest to the individual research subject are all topics a community member of the IRB evaluates. While the scientific and technical aspects of the study also demand inspection by the community member, the principal value of the community member's perspective lies in his or her contribution of local values, concerns, and culture.

With advances in genomic sequencing and the rise of precision medicine, all members of the IRB face a rapidly changing environment. While the changes in the scientific components of studies are extensive and complex, the potential impacts to patient protection are just as significant and dynamic. The traditional areas of primary concern, such as patient privacy, terminology, and financial benefits, are being joined by issues associated with precision medicine, such as accelerated FDA drug approval, multiple informed consent form modifications, increasing length and complexity of informed consent forms, and participant genetic privacy. The challenge to the IRB is to remain focused on those prior areas of significance while also adapting the evaluation process to the novel science of precision medicine.

In 2018, the FDA approved 59 novel drugs.^18^ As of May 24, the FDA had approved 11 new drugs in 2019.^19^ Of these, 6 are biological and address the needs of a narrow patient population. Five of the 6 received accelerated approval, orphan class, or breakthrough treatment designation. Some of these new entities also had therapy-specific screening tests approved. These 17 months of FDA activity illustrate the ever-changing environment that clinicians, principal investigators, and IRBs face. For investigators, this dynamic environment may require modifications to an existing protocol and informed consent form for an ongoing study comparing the standard of care to an investigational drug. For IRB members, this environment requires adapting processes and adjusting to the new technology and its corresponding complexities. Such adaptation has always been required of the IRB, but now it will need to occur rapidly as technology and data analysis capabilities accelerate.

### Precision Medicine Research Protocols and Informed Consent Forms

Changes to the Federal Policy for the Protection of Human Subjects—known as the Common Rule—that were effective January 21, 2019 started the process of updating the informed consent form to reflect the expansion of precision medicine and genomic analysis. However, these changes are only required for federally funded studies and therefore may not be applied to all consent processes. This discrepancy places the IRB in the position of having to decide if complying with the prior requirements is adequate for non–federally funded studies and raises the question of whether all studies should be required to conform to the revised Common Rule.

When the IRB is presented with a modification to a protocol and its informed consent form, the final decision to approve, deny, or require further modification depends on the impact of the modification in relation to the necessity of re-consenting the current research subjects. The ethical principle of respect for persons acknowledges that each person has the right to determine his or her own destiny.^20^ After careful review of the modifications requested, the IRB must decide if the changes need to be presented to the study participants in a new informed consent form and interview so that they are fully aware of the changes and the implications of those changes, whether positive or negative. The perspective “how would I like to be treated?” is invaluable to an IRB member. Typically, if procedures, risks, benefits, or duration of participation change, current participants need to be re-consented.

The development of new study designs—umbrella, basket, and adaptive trials—require IRBs to focus on some of the guiding principles of informed consent. A traditional study has design, conduct, and analyze phases. An adaptive study will have design, conduct, review, adapt, conduct (again), and analyze phases.^21^ Addition of review and adapt phases creates flexibility for the investigator and potential benefit for the participants in the study (such as pursuit of the most effective treatments and discontinuation of the least effective treatments) but requires a more complex informed consent form than a traditional study design uses. The IRB is charged with evaluating whether nonscientific participants can understand the informed consent form. As such, the consent document should be written in layman's terms*.*^22^ While the definition of lay language is open to multiple interpretations, ensuring potential research subjects’ understanding of what is presented in the informed consent form is the goal the IRB must strive for. Comprehension can be especially challenging if multiple processes and procedures are contained in a single consent form.

In addition to the challenge of comprehension, precision medicine protocols and their informed consent forms can be quite lengthy. Consent form length is a concern for the IRB and the investigator because the longer the informed consent process and form are, the more likely that potential study participants will miss key issues relevant to an informed decision.^23^ Participants may not bother to read the entire form or pay attention during a research coordinator's reading of the form. Concern about informed consent form length has been prevalent in the IRB and the literature for many years. Complexity, legal edits, Health Insurance Portability and Accountability Act requirements, and explanations of technical terms in precision medicine result in informed consent forms that exceed 30 pages in many instances. Revisions to the Common Rule addressed this dilemma somewhat by requiring a key information section at the beginning of the informed consent form. This new section is intended to provide a brief summary of the study and give subjects an overview of the design and components of the study to help with their decision about whether to participate. This summary addresses some concerns about fully informed consent, but it is only required in federally funded studies; industry- and investigator-sponsored studies need not comply with this requirement. The IRB must decide—based on the complexity of the study—whether the key information section should be required in a lengthy informed consent form.

### Genetic Privacy

Basket studies and umbrella studies rely on genomic testing for inclusion criteria, and umbrella studies rely on genomic testing for investigational drug therapy choices. The challenge to the IRB is to allow the sharing of participant genetic information for research while protecting participant privacy. One crucial concept is potentially damaging or embarrassing information about the research subject*.*^20^ The protection of privacy and confidentiality is central to the IRB's mission, so to protect participant privacy, the IRB must decide who is allowed to access the results of any genetic testing and the duration of such access. This issue is not restricted to researchers and external organizations but also applies to the participants themselves. Genetic information sharing raises questions such as these: If genetic testing reveals a mutation that is indicative of the possible development of a disease or syndrome, should the participant be given that information? Should the participant be given that information if no treatment is currently available for the disease or syndrome? Should the IRB anticipate the development in the future of a viable treatment? Will providing the information cause distress for the participant and therefore cause harm?

These questions have no standard answers. The IRB must evaluate each study and the participant population involved using the principle of respect for the individual and strive to keep research subjects as safe as possible.

### Coercion/Undue Influence

An aspect of the principle of respect for persons is the concept of coercion or undue influence. Coercion occurs when a person, to some degree, is forced, or at least strongly pushed, to do something that may not be in his or her best interest.^20^ In the field of oncology precision medicine research, many studies target specific genomic anomalies that have failed current standard of care treatment. The participant population that has run out of options could be susceptible to an overly optimistic perception of response to the investigational treatment. This perception is especially problematic in phase 1 studies that are designed to determine dosing and toxicity rather than assess therapeutic outcome. In such cases, the IRB is challenged with ensuring that the informed consent form is realistic in its risk-to-benefit discussion and clearly states the purpose of the study.

## CONCLUSION

In this era of precision medicine, new technologies, novel study designs, and innovative therapeutic entities will challenge and elicit change within the entire spectrum of clinical research. As precision medicine advances, the IRB will continue to confront new applications that require fresh approaches to the protection of research subjects. Input from all involved in the research process is necessary for the system to move forward and successfully navigate this evolving field.

## Notes

This article meets the Accreditation Council for Graduate Medical Education and the American Board of Medical Specialties Maintenance of Certification competencies for Patient Care, Medical Knowledge, and Systems-Based Practice.
